# A framework for assessing the economic impacts of Arctic change

**DOI:** 10.1007/s13280-019-01211-z

**Published:** 2019-06-24

**Authors:** Jimena Alvarez, Dmitry Yumashev, Gail Whiteman

**Affiliations:** 1 Pentland Centre for Sustainability in Business, Lancaster University, Lancaster, LA1 4YX UK; 2 Salguero 3055, 1425, Ciudad Autónoma de Buenos Aires, Buenos Aires, Argentina

**Keywords:** Arctic, Climate change, Economic impacts, Transdisciplinary science

## Abstract

The scientific literature on physical changes in the Arctic region driven by climate change is extensive. In addition, the emerging understanding of physical feedbacks and teleconnections between the Arctic and the rest of the world suggests that the warming in the Arctic region is likely to cause impacts that extend well beyond the region itself. However, there is only limited research on how Arctic change may affect economies and individual industry sectors around the world. We argue that there is a pressing need for more research on this topic and present a conceptual framework to guide future research for assessing the regional and global economic impacts of Arctic change, including both possible benefits and costs. We stress on the importance of a transdisciplinary approach, which includes an integration of the natural sciences, economics and social sciences, as well as engagement with a wide range of stakeholders to better understand and manage the implications of Arctic change.

## Introduction

The Arctic has been changing at unprecedented rates over the past three decades driven by climate change, with the average rate of warming in the region twice as high as the global average (IPCC 2013; Overland et al. 2015). The changes in the Arctic are manifested by the decline in the sea ice, permafrost, glaciers and the Greenland ice sheet (Stroeve et al. 2012; Van den Broeke et al. 2016; Chadburn et al. 2017).

In addition to the extensive scientific literature on physical changes in the Arctic region itself, there is an emerging scientific knowledge of physical feedbacks and teleconnections between the Arctic and the rest of the world (Burke et al. 2017; Francis et al. 2017). These physical processes will exacerbate the effects of climate change globally. Since climate change carries significant economic impacts worldwide (Stern 2007; Tol 2009; Hope 2013; Nordhaus 2013; Dietz and Stern 2014; IPCC 2014a, b; Burke et al. 2015), Arctic-driven feedbacks and teleconnections are expected to cause additional economic impacts far beyond the Arctic region itself (Whiteman et al. 2013; Hope and Schaefer 2016; Yumashev et al. 2019).

Yet economics research to date has focussed primarily on estimating economic opportunities due to Arctic change through increased oil and gas and mineral extraction, shipping, tourism and agriculture in the Arctic region (ACIA 2005; Gautier et al. 2009; Hovelsrud and Smit 2010; Hovelsrud et al. 2011; Emmerson and Lahn 2012; Smith and Stephenson 2013; Bekkers et al. 2018). More recently, multiple authors have recognised the potential negative economic impacts of Arctic change, both regionally in the Arctic and globally (Euskirchen et al. 2013; Whiteman et al. 2013; González-Eguino and Neumann 2016, 2017; Hope and Schaefer 2016; Melvin et al. 2017; Yumashev et al. 2019). Notably, Yumashev et al. (2019) assessed non-linear transitions in Arctic feedbacks driven by the loss of land permafrost, snow and sea ice covers, and estimated the resulting impacts on the global climate and economy under various climate mitigation scenarios. Despite this progress, the literature still lacks a comprehensive framework for assessing the costs and benefits of Arctic change. Without such a framework, policymakers could under or overestimate the true cost associated with Arctic change. This is the key gap that we wish to address here.

We argue that estimating the benefits and costs of Arctic warming requires a number of complementary methodologies and models, including specialised climate and ecosystem models, Integrated Assessment Models (IAMs), and both regional and global macroeconomic models. In other words, a transdisciplinary approach is required for understanding and managing the implications of Arctic change, which brings together natural sciences, economics, social sciences and engagement with a wide range of stakeholders (Whiteman and Yumashev 2018).

We build upon recent work in this area. For example, the European Union’s project Arctic Climate Change, Economy and Society (ACCESS) carried out a transdisciplinary assessment of physical impacts of climate change on the Arctic Ocean and the resulting socio-economic impacts within the Arctic region focussing on key economic activities: shipping, tourism, sea food production and natural resource extraction up to 2050 (NERC 2015; Crépin et al. 2017a; Gascard et al. 2017). A key contribution from the project is highly relevant to the issue at hand: the development of “a framework for integrated ecosystem-based management” (IEBM), which “accounts for complex interactions between society and nature, possible abrupt change, and substantial uncertainties” (Crépin et al. 2017a, b). Our proposed framework—though focussed on economics—extends the IEBM’s scope of analysis to account for the indirect global impacts from Arctic change and the secondary impacts through knock-on effects in the global economy.

The paper is structured as follows: “A framework for assessing the economic impacts from Arctic change” section introduces a framework for assessing the economic impacts from Arctic change and methods to appraise it; “Economic opportunities and regional impacts from a melting Arctic” section focusses on the economic benefits and direct regional impacts resulting from a melting Arctic; “Indirect global impacts via Arctic feedbacks and teleconnections, and secondary economic knock-on effects” section addresses the indirect global impacts from Arctic change, followed by concluding remarks in “Conclusion” section.

## A framework for assessing the economic impacts from Arctic change

### Framework

Given the global nature of Arctic climate feedbacks, the global economic costs of Arctic-related climate change may counter-balance the economic benefits from shipping, tourism, natural resource extraction and other industries enabled by a warming Arctic region. Thus, a key outstanding question is whether the changing Arctic could result in significant economic impacts worldwide, and if so, how best one could quantitatively assess these impacts over time.

**Fig. 1:**
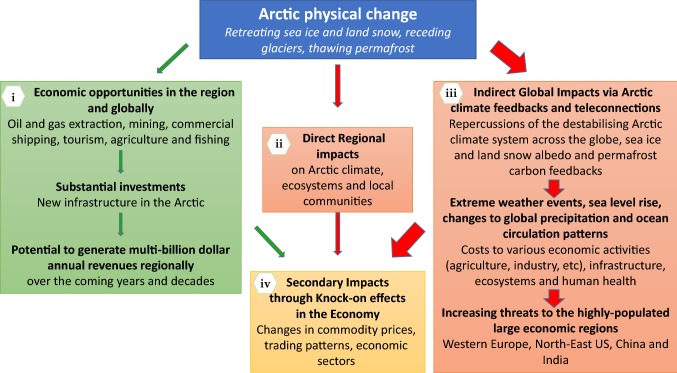
Benefits and costs of Arctic change: a holistic view. The width of the arrows represents the difference in impacts’ magnitude

Based upon existing literature from a variety of disciplines, Fig. 1 delineates how Arctic physical changes can trigger economic impacts—positive and negative—both on the regional and global levels. On the one hand, (i) new economic opportunities in the region associated with oil and gas and mineral extraction, commercial shipping, tourism, agriculture and fishing have the potential to generate multi-billion-dollar annual revenues (ACIA 2005; Gautier et al. 2009; Dyck and Sumaila 2010; Hovelsrud and Smit 2010; Hovelsrud et al. 2011; Emmerson and Lahn 2012; Lam et al. 2014; Bekkers et al. 2018). On the other hand, (ii) changes in the Arctic have direct regional impacts on its climate, ecosystems and communities (ACIA 2005; Hovelsrud et al. 2011; Wassmann et al. 2011; AMAP 2015b), (iii) as well as lead to indirect global impacts through Arctic climate feedbacks and teleconnections (Euskirchen et al. 2013; González-Eguino and Neumann 2016, 2017; Hope and Schaefer 2016; Yumashev et al. 2019). In addition, (iv) the revenues and impacts associated with Arctic change could result in secondary impacts through economic knock-on effects in multiple countries around the world (Countryman et al. 2016; Bekkers et al. 2018). Each of these four main components of Fig. 1 is discussed in the subsequent sections.

### Existing quantitative methods to assess Arctic change

The framework presented in this paper calls for more efforts towards estimating the extent and range of economic impacts associated with Arctic change. As explained in the previous section, by Arctic change we denote the impacts of global climate change manifested in the Arctic region. We argue that transdisciplinary science is crucial here since physical impacts often need to be translated into economic benefits and costs in order to engage with businesses and policymakers.

Each of the four main categories of impacts (benefits and costs) due to Arctic change, summarised in Fig. 1, requires different methodologies and models in order to perform quantitative assessment of the impacts. Estimating economic opportunities in the Arctic region and globally [category (i)] requires a combination of climate and ecosystem models and sector-specific impact models that translate changing climatic conditions into benefits and costs for each sector (Lam et al. 2014). The same applies to direct negative impacts in the Arctic region [category (ii)]. Assessing indirect global impacts of Arctic climate feedbacks and teleconnections (category (iii)) calls for IAMs calibrated according to the latest results from climate models and other specialised biophysical models (Yumashev et al. 2019). Finally, estimating secondary economic knock-on effects due to Arctic development requires regional and global macroeconomic models with interlinkages between multiple economic sectors (Bekkers et al. 2018), based on either general equilibrium or input–output methodologies.

On the climate modelling side, efforts to better understand the possible futures of Arctic sea ice, land and subsea permafrost and Greenland ice sheet, as well as their climatic impacts on other world regions, are ongoing. One particular difficulty is associated with the coupling of ice sheet and permafrost models with atmospheric, ocean and land models, which has not yet been attempted in the current generation of earth system models (CMIP5) that feature in IPCC AR5. Even before such coupling could be attempted, consensus must be reached on several underlying physical processes, most importantly a growth in the extreme weather events associated with volatile jet stream and emissions of methane from subsea permafrost.

On the economic modelling side, the growing literature on global economic impacts associated with climate change has relied on IAMs extensively (Hope 2013; IPCC 2014b), as well as direct econometric analysis of relationships between historic climate and economic data (Dell et al. 2012; Burke et al. 2015). The economic outputs from IAMs can help bridge the gap between climate science and policy (Ackerman and Stanton 2013), and provide a widely used methodology for assessing policy options under multiple uncertainties, which is achieved by combining simplified representations of the climate, economy and policy options (Parson and Fisher-Vanden 1997; Weyant and Hill 1999). Most climate policy studies based on IAMs employ the estimates of the regional and global costs of climate change represented as functions of the corresponding changes in mean annual temperatures and sea level. As a result, they do not include more sophisticated physical drivers such as changes in precipitation patterns and extreme weather events, and also tend to miss out on important climate feedbacks such as carbon emissions from thawing land permafrost in the Arctic. Another challenge for the IAMs like PAGE, DICE and FUND is to improve the so-called damage functions in order to provide a more defensible economic valuation of the indirect global impacts of Arctic climate feedbacks and teleconnections, and of the economic effect of climate change in general. Damage functions have been criticised for a variety of reasons, including their overall opacity and the high levels of uncertainty of the impacts at higher temperatures (Howard 2014; Pindyck 2017). The new IAM PAGE-ICE (Yumashev et al. 2019) addresses many of these challenges by including non-linear statistical representations of Arctic permafrost carbon feedback and sea ice and land snow albedo feedback based on complex physical models, and by employing empirical market damages by Burke et al. (2015) to estimate the associated global economic impacts.

Aside from IAMs, there is a need for specialised regional macroeconomic models for Arctic countries and states, such as Greenland, Alaska and Arctic parts of Canada and Russia, that are capable of translating sector-level impacts (Melvin et al. 2017) into secondary socio-economic effects in these areas. Regional studies from other parts of the world, for example a study by Crawford-Brown et al. (2013) on economic impacts of climate-driven flooding in London, have used input–output models. These models could be further enhanced to resolve secondary economic effects (both indirect and induced) of climate change in the Arctic countries and states by incorporating social accounting matrices (Yu et al. 2010). However, as with the estimates of global costs of Arctic change using IAMs, the biggest challenge for the regional economic assessments in the Arctic is to provide an accurate description of primary climate-driven economic impacts for each sector.

We acknowledge that uncertainty underpins climate change assessments both from scientific and socio-economic perspectives due to our incomplete knowledge (Heal and Millner 2014). For example, a recent study by Christensen et al. (2018) found that the IPCC’s RCP and SSP scenarios “miss the upper tail of productivity growth, implicitly understating the likelihood of high output growth rates and the resulting high emissions, concentrations, temperature change, and climate damages” (Christensen et al. 2018). Quantifying and ultimately narrowing down the uncertainties in the multiple physical and socio-economic processes associated with climate change, both in the Arctic and beyond, remains an important task for future research.

Finally, research suggests that ecosystem and biodiversity are crucial to the very existence of our societies, and hence measuring their worth in economic terms using ecosystem services has significant limitations (Costanza et al. 1997; IPBES 2019). Climate change poses a threat to these systems as “with increasing warming, some physical systems or ecosystems may be at risk of abrupt and irreversible changes” (IPCC 2014c); thus, global policymakers ought to seek appropriate ways of evaluation beyond the neo-classical economics framework. As an example, to depict the total value of the Arctic in the Earth system, economic impacts on their own would not suffice, and alternative methods such as multicriteria analysis could be worthwhile (Keeney and Raiffa 1993). We therefore acknowledge that a “social–ecological systems approach is required to better facilitate resilience-building, a key component of sustainable development” (Arctic Council 2016). Nevertheless, our understanding is that adapting both climate models, IAMs and macroeconomic models to include Arctic-specific effects and help estimate the associated economic costs (Yumashev et al. 2019) is a logical starting point towards highlighting the urgency of preventing the worst effects of Arctic change.

## Economic opportunities and regional impacts from a melting Arctic

### Economic opportunities

The large-scale physical changes that are underway in the Arctic are likely to lead to substantial investments into new infrastructure in the Arctic region, with the potential to generate multi-billion-dollar annual revenues over the coming years and decades (Emmerson and Lahn 2012). However, investment decisions in the Arctic are particularly difficult due to its restricted geographic access, environmental concerns, highly contrasting seasons and constrained markets, as well as the fact that many projects are transborder in nature since they include several Arctic states (WEF 2014), giving rise to sensitive geopolitical issues.

The short-term (years) and medium-term (until 2050) economic benefits of an Arctic change scenario include potential for oil and gas and mining exploration, increase in regional tourism, fishing, agriculture and commercial shipping to Arctic destinations (ACIA 2005; Gautier et al. 2009; Hovelsrud and Smit 2010; Hovelsrud et al. 2011; Lam et al. 2014), as well as medium- to long-term (beyond 2050) benefits from commercial shipping along transit Arctic routes (Hansen et al. 2016; Yumashev et al. 2017). An assessment by the United States Geological Survey of the area north of the Arctic Circle concluded “that about 30% of the world’s undiscovered gas and 13% of the world’s undiscovered oil may be found there, mostly offshore under less than 500 meters of water” (Gautier et al. 2009). In order to access these resources, substantial investment is needed: “except for certain areas of Norway and the western Russian Federation, the region remains vastly underserved by transportation, port and other critical infrastructure” (WEF 2014). Furthermore, a recent scenario-based study on the European Arctic Seas concludes that, even if oil and gas exploitation were possible from a technological point of view, “under current prices and with competing fossil and renewable energy sources, an exploitation does not seem to be rational from an economic point of view” (Petrick et al. 2017). The lack of infrastructure coupled with the remoteness of the region pose additional challenges to the management of potential oil spills (Harsem et al. 2011). In addition, the decrease in sea ice might result in “greater areal coverage and increased shoreline exposure” in future oil spills (Nordam et al. 2017). In a region where extreme weather increases the risk of an oil spill, a good starting point would be Greenland’s strategy of negotiating an upfront “clean-up bond” (Webb 2010; Harsem et al. 2011).

Climate change is a driver of ‘last-chance’ tourism in some Arctic locations, resulting in short- to medium-term benefits to local communities and tour operators in the region, which is a paradox considering that emissions associated with travelling to these remote locations tend to further reinforce the negative impacts of climate change (Lemelin et al. 2010). In addition, whilst sea ice decline could potentially increase cruise shipping in some Arctic regions (Dawson et al. 2014), a study based on a 37-year observational record in the Canadian Arctic stresses that hazardous sea ice conditions might prevent this from happening, at least in the near future (Stewart et al. 2007). Even for a modest increase of tourism in the region, infrastructure and regulatory modifications would be required (Lasserre and Têtu 2015).

A study on the impacts of climate change on the Arctic fisheries’ sector projects that total revenues may increase by 39% in 2050 vs. 2000 (33% when factoring in ocean acidification) which, in turn, is expected to have a positive “multiplier” effect of 3 on the whole Arctic economy (Dyck and Sumaila 2010; Lam et al. 2014). Positive impacts have already occurred such as the unprecedented arrival of the Atlantic mackerel in Greenland in 2011, which climbed from representing 0 in 2011 to 23% of its exports in 2014 (Jansen et al. 2016). On the other hand, the industrial fisheries might pose a threat to native Arctic marine fish species as it “turns up as unprecedented bycatch” (Christiansen et al. 2014). Hence, the extent to which Arctic fisheries will benefit from climate change is subject to a variety of factors: from the resulting socio-economic repercussions due to exploitation of the new species compositions to the risk posed by unsustainable fishing practices, particularly given the role some non-Arctic fishing countries with “more efficient and higher-powered fishing fleets”—such as Japan and China—might play in the region (Lam et al. 2014).

Even though Arctic change is enabling the development of agriculture in the region, some impediments still remain such as lack of infrastructure to promote commercial agriculture, water limitations, scant population, risk-averse behaviour of the farmers as well as inadequate governmental policies (ACIA 2005; Hovelsrud and Smit 2010; Hovelsrud et al. 2011). Even if climatic conditions were to enable enough agricultural produce to cover local demand and export the surplus, macroeconomic conditions are still likely to be the dominant factor. For instance, the competitiveness of prices might present an issue, in particular to the Arctic countries that are part of the European Union (ACIA 2005).

Medium- to long-term benefits of Arctic change also include shorter albeit inherently difficult transit shipping routes that could have a positive effect on the trade between Asia and Europe as well as between the East and West coasts of the US (Smith and Stephenson 2013; Aksenov et al. 2016; Hansen et al. 2016; Bensassi et al. 2016; Countryman et al. 2016; Bekkers et al. 2018). It has been estimated that around 5% of the world’s trade could be shipped through the Northern Sea Route (NSR) in the Arctic alone under a hypothetical year-round and unhampered navigability, generating additional income for many European and Asian countries (Bekkers et al. 2018). Despite the seemingly favourable near-term navigability trend dictated by sea ice retreat from NSR around the month of September in the coming decades (Aksenov et al. 2016), the shipping companies may delay investments in large-scale operations along NSR until profitability conditions are met (Hansen et al. 2016), which is likely to push the onset of large-scale commercial operations on NSR to the second half of the twenty-first century even under the worst-case scenarios in terms of the sea ice loss (Yumashev et al. 2017).

The changing Arctic, and its consequent effects on diverse economic sectors, has the potential to generate significant revenues. The extent to which such revenues materialise is subject to considerable uncertainty. A holistic approach which factors in the repercussions from economic development on the Arctic ecosystems and communities seems crucial to ensure a sustainable development of the Arctic region (Crépin et al. 2017b).

### Direct regional impacts from Arctic change

Without taking away the economic potential that could be unlocked by a warmer Arctic, one should acknowledge the likely negative impacts in the Arctic region itself as a result of the rapid climatic changes (IPCC 2014a, b). Climate impacts in the Arctic affect its ecosystems and influence the subsistence activities of local communities. These include impacts of thawing permafrost on local infrastructure, impacts from wildfires in tundra and boreal forests, and changes in wildlife and plant species distribution patterns (ACIA 2005; Higuera et al. 2008; Hovelsrud et al. 2011; Mack et al. 2011; Melvin et al. 2017). According to AMAP’s latest assessment on human health in the Arctic: “The most pronounced impacts of climate change in the Arctic occur in small communities in regions with infrastructure dependent on permafrost stability and where ice is needed for travel, hunting and the protection of the shoreline from coastal erosion.” (AMAP 2015b, p. 137).

Several areas around the Arctic Ocean were identified as high-risk potential hazard of thawing permafrost within the Northern Hemisphere (Nelson et al. 2001). Thawing permafrost can lead to several negative effects: “threatens coastal settlements; damage to poorly engineered and constructed infrastructure; release of legacy pollutants that affect the food chain and have negative health effects; tree death caused by drought; increased forest fire occurrence” (Hovelsrud et al. 2011). Socio-economic impacts of thawing permafrost include damages to infrastructure. Even though the number of settlements in the Arctic tundra is below 400 and most of them are relatively small, some Russian cities in the region exceed 100 k population (Streletskiy et al. 2015). With a tendency of Arctic settlements to be located in coastal areas, an increase in coastal erosion might force settlements to relocate (Streletskiy et al. 2015). A study in Prudhoe Bay Oilfield in Alaska—the first oilfield which was developed in the Arctic in ice-rich permafrost (IRP) terrain—showed a doubling in flooding and more than tripling in thermokarst across a number of areas in the period between 1980 and 2010 (Raynolds et al. 2014, Fig. 6). With the prospect of continued negative impacts from thawing permafrost on infrastructure, mitigation strategies like thermosiphons could offer a valuable coping mechanism (Streletskiy et al. 2015).

According to ACIA (2005)’s report: “Large-scale forest fires and outbreaks of tree-killing insects are characteristic of the boreal forest, are triggered by warm weather, and promote many important ecological processes”. For example, in 2007 over 1000 km^2^ of Arctic tundra were burnt in the Anaktuvuk River fire in Alaska, “doubling the cumulative area burned in this region over the past 50 years” (Mack et al. 2011). Thawing of permafrost may increase the risk of late season fires—such as those in the Anaktuvuk river basin—in tundra regions (Hu et al. 2010). In addition to the potential release of significant amounts of organic carbon, another impact of increased fires is the change in vegetation from graminoid to shrub tundra which, in turn, could further reinforce climate change (Mack et al. 2011). Based on a study of paleorecords in Alaska, Higuera et al. (2008) implied that “ongoing shrub expansion and climate warming will result in greater burning within northern tundra ecosystems”.

A review of over 50 reports on the effects of climate change on Arctic marine ecosystems concludes that there is “compelling evidence of impacts of climate change on almost all components of the marine ecosystems” and further stresses that it is likely that many other impacts have not been documented yet (Wassmann et al. 2011). A global projection of climate change impacts on a sample of 1000 + marine species identifies the Arctic as one of two regions with the highest species turnover by 2050 (Cheung et al. 2009). The potential development of commercial shipping routes through the Arctic could result in an increase of marine species invasion in the region (Whitman Miller and Ruiz 2014). In addition, under continued warming, the Bering Strait could enable the passage of mollusks and other species from the Pacific to the Atlantic Ocean (Vermeij and Roopnarine 2008).

Despite the possible economic benefits from Arctic shipping and oil and gas extraction, a recent study along the Norwegian coast suggests that local emissions from oil and gas and shipping are already impacting air pollutant levels in the region [ozone and aerosols such as sulphates and black carbon (BC)] (Law et al. 2017). Furthermore, a substantial increase in Arctic shipping and oil and gas extraction is expected to lead to higher environmental risks from short-lived pollutants such as BC, as well as oil spills (Harsem et al. 2011; AMAP 2015a). For example, the projected increase in shipping traffic along the NSR could result in a total climate feedback contribution of “0.05% (0.04%) to global mean temperature rise by 2100 under the RCP8.5 (RCP4.5) climate change scenario”, offsetting the economic gains from shipping by a third and a quarter, respectively (Yumashev et al. 2017).

Econometric analysis is crucial for assessing both the benefits and direct economic impacts in the Arctic region. The latest Economy of the North (ECONOR) 2015 report presents an overarching analysis of the circumpolar Arctic economy for 2006–2012, building up on the ECONOR 2006 and 2008 reports (Glomsrød and Aslaksen 2006, 2009; Glomsrød et al. 2017). The report highlights that climate change impacts on the Arctic economy are mostly concealed by other effects at the macro-level perspective of the report. The use of a common format for expressing the data constitutes a significant improvement vs. previous studies which enables comparison between Arctic regions as well as within Arctic countries (Glomsrød et al. 2017). For instance, most Arctic regions—except Finland, Sweden and Norway—had higher gross regional product (GRP) per capita as well as disposable income of household per capita in 2012 than their non-Arctic counterparts (Glomsrød et al. 2017). The sectoral analysis by region depicts great variability: with Arctic Russia, Alaska and Northern Canada leading on petroleum and other mineral extraction whilst Greenland and the Faroe Islands’ economies focus towards natural resources and secondary industries take the lead in Sweden and Finland (Glomsrød et al. 2017).

The socio-economic diversity within the Arctic region underscores the need for detailed Arctic econometric analysis—instead of national values—within models to get useful and more realistic results. The report also highlights the need to develop “satellite accounts” for production and consumption data on subsistence activities, something almost unaccounted for except for Alaska, following the United Nations recommendation (Glomsrød et al. 2017).

The report also includes a pilot study on the current anthropogenic impact on (the Arctic county) Finmark’s biodiversity using the GLOBIO 3 model, which “estimates biodiversity loss by measuring the impact of different pressures based on cause–effect relationships derived from research literature” (Glomsrød et al. 2017). Even though the focus of the analysis is on current impacts, which need to be extended for future and/or past scenarios, it is a useful depiction of the adjustments needed to adapt the model data for conducting analysis at the local level. Conducting similar pilot studies on different areas and regions, and coupling it with future scenario analysis, could be a useful tool for shaping policy in the Arctic region.

A recent study by Anisimov et al. (2017) uses predictive modelling to assess the climate change impacts on Arctic ecosystem services under the RCP 8.5 scenario. Their analysis entailed developing Arctic-specific outlets such as a “detailed digital vegetation map” and “statistical vegetation model” as well as constructing an ensemble of CMIP5 Earth System models which consisted of the best model fits for Arctic regional performance.

All of these impacts add to the stresses that Arctic ecosystems and local communities are subject to from the rapidly changing regional climate. Even though Arctic communities have a track record of high adaptability to natural variability, “the rate and magnitude of such changes represent unprecedented challenges to the current adaptive capacity and resilience of Arctic residents” (Keskitalo et al. 2010; Hovelsrud et al. 2011). There is an urgent need to put socio-economic policies in place that will help Arctic communities adapt to climatic changes in the region.

## Indirect global impacts via Arctic feedbacks and teleconnections, and secondary economic knock-on effects

### Indirect global impacts

The rapid warming in the Arctic region is of global concern due to a number of Arctic-driven feedbacks and teleconnections, including an increase in global sea level rise from the melting of the Greenland ice sheet (Chylek et al. 2009; Tedesco et al. 2011; Francis and Vavrus 2012), greenhouse gas emissions from thawing permafrost on land (Schuur et al. 2009, 2015; Schaefer et al. 2011) and subsea (Romanovskii et al. 2005; Shakhova et al. 2010, 2014, 2017; Nicolsky et al. 2012), increased solar absorption in the Arctic Ocean due to sea ice and snow retreat (Flanner et al. 2011), increase in ocean acidification (Bates and Mathis 2009), changes to global precipitation patterns (Givati and Rosenfeld 2013), and growing extreme weather events attributed to increased jet stream volatility (Cohen et al. 2014; Coumou et al. 2014; Hall et al. 2015; Francis and Vavrus 2015; Kug et al. 2015; Francis et al. 2017). There is also an added risk of changes to the North Atlantic Ocean circulation due to freshwater discharge from the Greenland ice sheet (Golledge et al. 2019). These processes have accelerated dramatically over the past three decades and have the potential to affect the overall stability of the climate system both in the Arctic, in the entire northern hemisphere and globally (IPCC 2013).

The magnitudes of these effects and the extent to which at least some of them stem from Arctic change are under debate (Barnes and Screen 2015; Francis and Vavrus 2015; Sapart et al. 2017). For instance, the possible link between Arctic warming and an increase in extreme weather events in mid-latitude regions would affect various economic sectors in Europe, North America and Asia, including agriculture, tourism and insurance (Francis et al. 2017). To put this in a perspective, global annual weather-related losses increased from around US$ 50 billion in 1980 to around US$ 150 billion in 2012 (Munich Re 2013; The World Bank Group Experience 2013), although a significant part of this increase has been attributed to socio-economic factors alone (Bouwer 2011; Mohleji and Pielke 2014).

Arctic climate feedbacks that carry economic costs globally include methane emissions from thawing permafrost. CO_2_ and methane releases from land-based permafrost represent another potential threat (Schuur et al. 2015; Burke et al. 2017), and economic estimates suggest that the associated cost to global economy could be around 40 trillion dollars over the next two centuries (Hope and Schaefer 2016). Euskirchen et al. (2013) estimate that “Between 2010 and 2100, the annual costs from the extra warming due to a decline in albedo related to losses of sea ice and snow, plus each year’s methane emissions, cumulate to a present value cost to society ranging from US$7.5 trillion to US$91.3 trillion”.

Most recently, Yumashev et al. (2019) used state of the art permafrost models and climate models of the current generation (CMIP5) to explore non-linear transitions in the climate feedbacks associated with the loss of Arctic land permafrost, snow and sea ice, and estimate the resulting impacts on global climate and economy. Introducing statistical emulators of the physical models in a new IAM PAGE-ICE, the authors found that the global economic impact of the Arctic feedbacks could reach $67 trillion over the next three centuries under long-term mitigation levels consistent with current national pledges (NDCs). This figure drops, respectively, to $34 and $25 trillion for the 2 °C and 1.5 °C target scenarios from the Paris Agreement, which further advocates for pursuing ambitious mitigation efforts to limit the extent of climate change to well below 2 °C from pre-industrial.

In addition, an earlier study assessed one of the more extreme scenarios, for example, which could occur when warming Arctic waters lead to the abrupt atmospheric release of methane from gas hydrates which are stored under the subsea permafrost on the Arctic shelf (Shakhova et al. 2010). This worst-case scenario could cost the global economy an estimated $60 trillion over the next two centuries (Whiteman et al. 2013). Whilst some natural scientists suggest that such sudden releases of vast quantities of methane are implausible (e.g. Archer 2015), others argue that underwater methane release in the East Siberian Sea is a valid threat (Romanovskii et al. 2005; Nicolsky et al. 2012; Shakhova et al. 2017). More research is required to unravel such complex and understudied issues.

### Knock-on effects on the economy

In addition to climatic feedbacks and teleconnections associated with Arctic change, economic developments in the Arctic region itself are likely to generate various costs and benefits globally through knock-on effects in the economy. These are manifested by Arctic-driven shifts in commodity prices and trading patterns, potentially leading to changes in economic sectors and social welfare in multiple countries around the world. It is a new field of research and there are very few relevant impact studies available, mostly concerning Arctic shipping. Bekkers et al. (2018) estimate that year-round navigability on NSR could increase the trade between EU and Asia by up to 6%, resulting in a 0.14% higher GDP in China, a 0.12% higher GDP in the EU (Belgium is the biggest winner among the EU countries with a 0.4% increase in the GDP), 0.15% in Japan and 0.23% in South Korea. However, as mentioned earlier, the potential economic gains from increased shipping along the NSR may be offset partially by the climate-related costs from the associated changes in the GHG emissions (climate feedback of the NSR), with most of the climate costs expected to occur in the poorer regions such as Africa and India (Yumashev et al. 2017). As is the case with all the other types of impacts of Arctic warming, the assessments of the knock-on economic effects are also characterised by uncertainties. These stem from the inherent uncertainty in the general equilibrium and input–output models, the commonly used tools for such assessments.

The policy implications of both the indirect impacts from Arctic change and its knock-on effects on the economy are more intricate than those from economic benefits and direct impacts in the Arctic region itself. From a modelling analysis perspective, our understanding is that replicating the types of analysis of the regional costs and benefits in the Arctic discussed in “Economic opportunities and regional impacts from a melting Arctic” section, coupled with scenario analysis of both different climatic and socio-economic drivers, could be useful to shape future policies targeting a wide range of sectors within Arctic economies. On the other hand, studies on the global impacts from Arctic change could be useful to inform policy initiatives at the regional and global level given that the climate change impacts in the Arctic region act as a barometer of the likely global climate impacts in the coming years. Furthermore, research so far indicates the magnitude of the global economic impacts of Arctic change is likely going to exceed that of the impacts in the Arctic region itself, particularly when it comes to additional climate losses due to Arctic feedbacks. This puts further pressure on governments around the world to adopt ambitious mitigation policies.

## Conclusion

The rate of Arctic change in the recent years causes negative impacts on climate, ecosystem and communities that extend well beyond the Arctic region (Bates and Mathis 2009; Shakhova et al. 2010, 2014; Givati and Rosenfeld 2013; Coumou et al. 2014; Hall et al. 2015). Existing research has focussed primarily on estimating economic impacts (usually opportunities) in the Arctic region itself (ACIA 2005; Gautier et al. 2009; Emmerson and Lahn 2012; Smith and Stephenson 2013). However, given the direct physical relationship between Arctic change and the global climate system, economic impacts are not likely restricted solely to the Arctic region.

In this paper, we presented a new framework for an economic assessment of both regional and global impacts of Arctic change that could help advise businesses and policymakers. There have been several studies attempting to quantify some of these impacts in economic terms (Euskirchen et al. 2013; Whiteman et al. 2013; Lam et al. 2014; Hope and Schaefer 2016; Yumashev et al. 2019), and we argue that a transdisciplinary approach with strong integration of climate science, economics and policy studies is required. The new framework encourages a more balanced perspective on Arctic development, and both on regional and global risks associated with Arctic change.

Arctic change can cause socio-economic impacts both at the regional and global levels. Growing industrial activities in the region are closely related to negative environmental impacts, for example black carbon pollution from shipping and greater risks of oil spills (Harsem et al. 2011; AMAP 2015a). Local Arctic communities thrive from the natural resources available in the region and hence climatic changes bring about new threats. Thawing permafrost poses a risk to existing infrastructure and requires adaptation of certain traditional activities—like hunting. A side effect of thawing permafrost is the potential release of contaminants held in the frozen soil (AMAP 2015b). Since Arctic change poses a threat to food and water security for Arctic communities, there is a need for monitoring programmes comprising quantitative indicators (Nilsson et al. 2013). On the global level, the extent of Arctic-related effects is highly uncertain but could cause multiple losses associated with rising sea level from the melting Greenland ice sheet, additional carbon emissions from thawing permafrost, additional warming due to the loss of the sea ice and snow covers and growing extreme weather events due to increased polar jet stream volatility. In addition, the limits to adaptation funds and/or political unwillingness to invest in mitigation could lead to political and economic tipping points both in the Arctic region and globally (Huntington et al. 2012).

Given the global and systemic nature of Arctic climate feedbacks, the associated economic costs may counter-balance and possibly outweigh the economic benefits arising from a warming Arctic region. A comprehensive framework for assessing the total economic effect of Arctic change presented here could help guide both individual investment decisions associated with Arctic change, and a wider climate policy.
